# Interactions of the Gasotransmitters Contribute to Microvascular Tone (Dys)regulation in the Preterm Neonate

**DOI:** 10.1371/journal.pone.0121621

**Published:** 2015-03-25

**Authors:** Rebecca M. Dyson, Hannah K. Palliser, Joanna L. Latter, Megan A. Kelly, Grazyna Chwatko, Rafal Glowacki, Ian M. R. Wright

**Affiliations:** 1 Mothers and Babies Research Centre, Hunter Medical Research Institute, New Lambton Heights, NSW, 2305, Australia; 2 School of Medicine and Public Health, University of Newcastle, Callaghan, NSW, 2308, Australia; 3 Illawarra Health and Medical Research Institute and Graduate School of Medicine, University of Wollongong, NSW, 2522, Australia; 4 School of Biomedical Sciences and Pharmacy, University of Newcastle, Callaghan, NSW, 2308, Australia; 5 Department of Environmental Chemistry, Faculty of Chemistry, University of Lodz, 90–236, Lodz, Poland; 6 Kaleidoscope Neonatal Intensive Care Unit, John Hunter Children’s Hospital, New Lambton Heights, NSW, 2305, Australia; Center for Cancer Research, National Cancer Institute, UNITED STATES

## Abstract

**Background & Aims:**

Hydrogen sulphide (H_2_S), nitric oxide (NO), and carbon monoxide (CO) are involved in transitional microvascular tone dysregulation in the preterm infant; however there is conflicting evidence on the interaction of these gasotransmitters, and their overall contribution to the microcirculation in newborns is not known. The aim of this study was to measure the levels of all 3 gasotransmitters, characterise their interrelationships and elucidate their combined effects on microvascular blood flow.

**Methods:**

90 preterm neonates were studied at 24h postnatal age. Microvascular studies were performed by laser Doppler. Arterial COHb levels (a measure of CO) were determined through co-oximetry. NO was measured as nitrate and nitrite in urine. H_2_S was measured as thiosulphate by liquid chromatography. Relationships between levels of the gasotransmitters and microvascular blood flow were assessed through partial correlation controlling for the influence of gestational age. Structural equation modelling was used to examine the combination of these effects on microvascular blood flow and derive a theoretical model of their interactions.

**Results:**

No relationship was observed between NO and CO (p = 0.18, *r* = 0.18). A positive relationship between NO and H_2_S (p = 0.008, *r* = 0.28) and an inverse relationship between CO and H_2_S (p = 0.01, *r* = -0.33) exists. Structural equation modelling was used to examine the combination of these effects on microvascular blood flow. The model with the best fit is presented.

**Conclusions:**

The relationships between NO and H_2_S, and CO and H_2_S may be of importance in the preterm newborn, particularly as NO levels in males are associated with higher H_2_S levels and higher microvascular blood flow and CO in females appears to convey protection against vascular dysregulation. Here we present a theoretical model of these interactions and their overall effects on microvascular flow in the preterm newborn, upon which future mechanistic studies may be based.

## Introduction

Endogenous hydrogen sulphide (H_2_S) is associated with microvascular tone regulation at 24h postnatal age in the preterm infant and production appears to be affected by both gestational age and sex [1]. Nitric oxide (NO) and carbon monoxide (CO) also play a crucial role in the transitional circulation of preterm neonates [2,3]. NO is proposed to play a central role in the maintenance of vascular homeostasis in the perinatal period, however urinary excretion of NO metabolites do not correlate with early changes in microvascular blood flow in the preterm neonate [4]. It has been hypothesised that the rate of NO production in the endothelium of peripheral microvessels (via endothelial nitric oxide synthase (eNOS)) is lower than would be required to activate the downstream sGC pathway in vascular smooth muscle cells responsible for the excessive vasodilatation seen in premature neonates. This has led to the speculation that other mechanisms may be involved in both the production of NO in the microvasculature and its vasoactive effects on vascular smooth muscle cells during the transition from fetal to neonatal circulatory systems, with NO contributing to the maintenance of background tone throughout this period [5,6]. CO levels, on the other hand, correlate with both gestational age and microvascular blood flow at 24h postnatal age, suggesting that CO production by very preterm neonates may contribute to their increased risk of microvascular dysfunction and physiological instability [4].

The interaction of these gasotransmitters may account for a large proportion of their action. For example, NO and CO interact in the neonatal cerebral vasculature to regulate vascular tone—acute elevation in CO produces vasodilatation, yet prolonged production inhibits NO production, causing cerebrovascular constriction. Knecht et al. [7], hypothesised this interaction between CO and NO may form the basis of a negative feedback system in the control of cerebrovascular tone. However, the interaction between NO and CO, and between these two systems and H_2_S may not be as simple as this. A number of studies examining the relationship between the gasotransmitters have been published, with conflicting results. The different findings reported by these various studies may be due to differences in the tissues studied, the animal studied and/or their developmental stage and the methods used [8,9,10]. Some of these findings are summarised in Table 1. Whilst this is not an exhaustive list of all studies examining the interactions between the gasotransmitters, it gives some idea of the wide-ranging results observed in a number of discrete studies.

**Table 1:** Published Interactions of the Gasotransmitters.

****Effector****	****Interaction****	****Tissue****	****Species****	****Developmental Stage****	****Reference(s)****
*Nitric Oxide*					
	↑ HO-1 expression (protein)	Aortic endothelial cells	Bovine		[11]
	↑ CO production and action	Cerebral vessels (pial arterioles)	Pig	Neonatal	[12,13,14]
	↑ CO action (permissive enabler)	Retina	Salamander		[15]
	↑ CO, ↑ HO-1 expression (protein, mRNA)	Aortic smooth muscle cells	Rat		[16]
	↑ CO, ↑ HO-1 expression	Mesangial cells	Rat		[17]
	↑ CO, ↑ HO-1 expression	Fibroblasts (from lung)	Human	Embryonic	[18]
	↑ CO, ↑ HO-1 expression	Kidney epithelial cells (cell line: LLC-PKI)	Pig	Juvenile (male)	[19]
	↑ CO, ↑ HO-1 expression	Macrophages (cell line: RAW264.7)^1^	Mouse		[20]
	↓ CO (via HO-1 inhibition)	Purified proteins	Human		[21]
	↓ HO activity	Aortic endothelial cells (cell line: AG08472)	Pig		[22]
	↓ HO-2 activity	Purified proteins	Rat		[23]
	↑ CSE expression	Peritoneal macrophages	Mouse	Adult (male)	[24]
	↑ H_2_S, ↑ CSE expression	Aorta	Rat	Adult (male)	[25]
	↓ CBS activity	Purified Proteins	Human		[26,27]
*Carbon Monoxide*					
	↑ NO release	Pulmonary artery endothelial cells	Bovine		[28]
	↑ NO release (at low concentrations of CO)	Renal arteries	Rat	Adult (male)	[29]
	↓ NO, ↓ eNOS (at high concentrations of CO)	Renal arteries	Rat	Adult (male)	[29]
	↓ NO (via NOS inhibition) after prolonged elevation of CO	Cerebral vessels (pial arterioles)	Pig	Neonatal	[7]
	↓ NO (via NOS inhibition)	HO-1, HO-2 constructs	Rat		[23]
	↓ NOS	Cerebellum (granule cells)	Rat	Neonate	[30]
	↓ iNOS activity, ↓ nNOS activity	Macrophages (iNOS), cerebellum (nNOS)	Rat		[31]
	↓ iNOS expression (transcriptional level)	Astrocytes	Human	Fetal	[32]
	↓ nNOS activity	Cerebellum (granule cells)	Rat	Neonatal	[33]
	↓ H_2_S (via CBS inhibition)	Astrocytes	Mouse	Neonatal	[34]
	↓ H_2_S, ↓ CSE expression	Aortic smooth muscle cells	Rat	Juvenile (male)	[35]
	↓ H_2_S (via CSE inhibition)	Carotid body	Mouse, rat	Adult (male)	[36]
*Hydrogen Sulphide*					
	↑ NO release	Brain homogenates	Rat		[37]
	↑ NO action (permissive enabler)	Ileum, aorta	Guinea Pig, Rat	Juvenile	[38,39]
	↓ NO effect	Aorta	Rat		[40]
	↓ NO	Retina	Salamander		[15]
	↓ NO activity	Aorta	Rat	Adult (male)	[41]
	↓ NO, ↓ iNOS	Macrophages (cell line: RAW264.7; lipopolysaccharide exposed)^1^	Mouse		[42]
	↓ eNOS	Aorta	Mouse, rat	Juvenile (male)	[43]
	↓ eNOS, ↓ nNOS, ↓ iNOS	Recombinant proteins			[44]
	↑ CO, ↑ HO-1 expression (protein, mRNA)	Pulmonary arteries (with induced hypoxic pulmonary hypertension)	Rat	Juvenile (male)	[45]
	↓ CO, ↓ HO-1 expression (protein)	Aortic smooth muscle cells	Rat	Juvenile (male)	[35]

HO heme oxygenase; CSE cystathionine-γ-lyase; CBS cystathionine-β-synthase; NOS nitric oxide synthase (eNOS endothelial isoform, iNOS inducible isoform, nNOS neuronal isoform). ^1^leukaemic monocyte macrophage cell line.

The synergistic effect of the interactions between these gasotransmitters arises from their different signalling cascades and their ability to enhance or diminish the effects of one or more of the others. The aim of the present study was to establish theoretical models of the interactions of the gasotransmitters and their combined effects on blood flow in the preterm newborn. This would provide a framework for establishing and testing current and future mechanistic hypotheses in this population. In order to achieve this, we measured the levels of all three gasotransmitters in the one neonatal population and characterised the interrelationships between NO, CO and H_2_S using structural equation modelling. As the differences in CO and H_2_S independently only account for a proportion of the vascular dysfunction observed in preterm neonates at 24h postnatal age we hypothesised that the interactions of NO, CO and H_2_S would account for a greater proportion of the microvascular tone dysregulation observed in the preterm newborn than the investigation of each of these molecules in isolation.

## Methods

### Subjects

Neonates 24–36 weeks’ gestation (n = 96) were studied at 24h postnatal age as part of the Cardiovascular Adaptation of the Newborn Study 2 (CANS2). These neonates form part of the cohort reported on previously [1,46]. Hypoxic ischemic encephalopathy, congenital malformations, chromosomal disorders or known congenital infection excluded admission to this study. The study protocol was approved by the human ethics committees at John Hunter Hospital and the University of Newcastle. Parental informed, written consent was obtained prior to investigation.

### Microvascular studies

Investigations were performed at 24h postnatal age with a Periflux 5001 laser Doppler (Perimed AB, Jarfalla, Sweden) with a temperature-regulated probe (Probe 457, Perimed) set at 36°C sited on the lateral aspect of the neonates’ lower limb as previously described [46].

### Clinical Illness severity

Clinical illness severity was evaluated using the Clinical Risk Index for Babies (CRIB) II scoring system [47].

### Carbon monoxide measurement

CO binds competitively to haemoglobin, in preference to oxygen, to form carboxyhaemoglobin (COHb), which represents an in vivo sink for CO. Arterial COHb levels were determined at 24h postnatal age through spectrophotometry by using an ABL700 blood gas analyzer (Radiometer, Copenhagen, Denmark) and expressed as a proportion of total haemoglobin concentration as previously described [4].

### Urine collection

24-hour urine samples were collected on day 2 of postnatal life as previously described [48]. Exact 24-hour urinary output was calculated by weighing diapers before and after use. As humidity can contribute to diaper weight, the degree and length of time in humidity were recorded and adjustments were made to calculate “true” increase as previously described [49].

### Nitrate/nitrite measurement

NO has a short physiological half-life, making it difficult to measure directly. In order to assess total body turnover of NO, the more stable end products of NO oxidation, nitrate and nitrite, were measured in urine using a commercially available colorimetric assay according to manufacturer’s instructions (Cayman Chemical Company, Ann Arbor, USA). Nitrate/nitrite levels were adjusted for 24h output and body weight to give a measure of total body output/24h (nmol/24h/kg).

### Thiosulphate measurement

Thiosulphate, a stable urinary metabolite of H_2_S was used to assess total body turnover of H_2_S, due to the short half-life and volatile nature of the gas. Thiosulphate was measured by reversed-phase liquid chromatography as previously described [1,50]. Thiosulphate levels were adjusted for 24h output and body weight to give a measure of total body output/24h (nmol/24h/kg).

### Statistical methods

Stata 13 for MacOSX (StataCorp LP, Texas, USA) was used for statistical analyses and structural equation modelling. Stata 13 and Prism 6 for MacOSX (GraphPad Software Inc., La Jolla, CA) were used for generation of figures. Data are presented as median (range) or mean and SEM where appropriate. Differences between groups were analysed by Mann-Whitney U-test unless otherwise stated. The relationships between levels of CO, NO, H_2_S and microvascular blood flow were assessed through partial correlation controlling for the influence of gestational age as in our previous studies [1,4]. Structural equation modelling was then performed in order to examine the combination of these effects on microvascular blood flow.

Structural equation modelling allows the examination of complex causal hypotheses on a set of intercorrelated non-experimental data and can be used for both exploration and confirmation of theoretical models [51]. For an exploratory approach such as that presented in the current study, a detailed model specifying the relationships among variables is not made *a priori*. This approach is considered superior over other correlational methods such as regression as multiple variables are analysed simultaneously, and latent factors reduce measurement error. When used as an exploratory or confirmatory approach, structural equation modelling provides information about the complex nature of disease and health behaviours. This is achieved by the examination of both direct and indirect, and unidirectional and bidirectional relationships between measured and latent variables [52]. In our particular construct, this was the interaction between the three gasotransmitters and their individual and combined effects on microvascular blood flow. All possible models were manually constructed for our three input (NO, CO and H_2_S) and one output (microvascular blood flow) variables. These models were then tested and assessed for suitability by χ^2^ Goodness of Fit and root mean square error of approximation (RMSEA). Lower χ^2^ values represent a better predicted model, whilst an RMSEA of below 0.06 shows a good fit [53]. RMSEA also allows for the calculation of a confidence interval (CI) around the predictive value of the model [54].

## Results

Of the 96 preterm neonates in the cohort, 6 neonates did not have complete data and could not be included in the model. Physical and clinical characteristics, including microvascular blood flow measurements for the remaining 90 neonates included in the model are reported in Table 2.

**Table 2:** Physical Characteristics of Neonates.

	Female (n = 43)	Male (n = 47)
Gestational Age (weeks)	28 (24–35)	29 (24–35)
Birth Weight (kg)	1.06 (0.45–2.38)	1.27 (0.56–2.76)
Microvascular Blood Flow (PU)	43.4 (4.7–266.8)	40.4 (6.5–216.64)
Completed Antenatal Glucocorticoids (n, %)	31 (72%)	36 (77%)
APGAR score at 5 min	8 (4–10)	9 (4–10)
Clinical Risk Index for Babies II score	8 (0–15)	5 (0–16)
Mean Blood Pressure at 24h	37.5 (24–68)	38 (26–81)
Small for Gestational Age	1 (2%)	6 (13%)
Significant patent ductus arteriosus	11 (26%)	12 (26%)
Intraventricular haemorrhage, grade ≥2	2 (5%)	3 (6%)
Sepsis	11 (26%)	13 (28%)
Died	4 (9%)	4 (9%)

Data presented as median (range) or number (percentage) as appropriate. PU laser Doppler perfusion units

Consistent with our previously reported observations in this cohort of neonates, which included the 96 preterm neonates (as well as 42 term neonates)) [46], microvascular blood flow at 24h postnatal age correlated with gestational age in this subset of neonates (all neonates p<0.0001, *r* = -0.54; females p = 0.009, *r* = -0.41; males p<0.0001, *r* = -0.64). There was no effect of birth weight on baseline microvascular blood flow when gestational age was accounted for (all neonates p = 0.82, *r* = -0.03; females p = 0.36, *r* = -0.15; males p = 0.66, *r* = 0.07). Detailed microvascular blood flow data for this cohort of neonates has been previously published [46].

### Gasotransmitter measurement

NO levels were higher in females than males (females 21.4(4.6–37.1)nmol/24h/kg vs males 20.1(0.9–56.7)nmol/24h/kg, p = 0.058). No differences in CO levels between sexes were observed (females 1.5(0.9–4.1)% vs. males 1.4(0.9–2.2)%, p = 0.29). Thiosulphate levels have been previously reported for this cohort [1].

### Interactions of the Gasotransmitters and their relationship with microvascular blood flow

Gestational age at birth was related to total gasotransmitter levels: gestational age was inversely correlated with NO (p = 0.03, *r* = -0.24), with no differences observed between males and females. Gestational age was also inversely correlated with CO (p = 0.0003, *r* = -0.45) and H_2_S (p = 0.02, *r* = -0.25). As urinary nitrates and thiosulphate are standardised to body weight, we could not examine the relationship between birth weight and NO and H_2_S. For CO, there was no effect of birth weight when gestational age was accounted for (all neonates p = 0.21, *r* = -0.17; females p = 0.14, *r* = -0.17; males p = 0.42, *r* = -0.15).

As gestational age is strongly related to the level of gasotransmitters, the relationships between levels of CO, NO, H_2_S and microvascular blood flow were assessed through partial correlation, controlling for the influence of gestational age. We observed a significant positive relationship between NO levels and microvascular blood flow for males but not females (p = 0.03, *r* = 0.38; females p = 0.91, *r* = -0.02). Furthermore, there was a significant positive relationship between CO levels and microvascular blood flow for males but not females (males p = 0.03, *r* = 0.38; females p = 0.88, *r* = -0.03) and H_2_S and flow (males p = 0.05, *r* = 0.29; females p = 0.72, *r* = 0.06).

Again, using partial correlations in order to account for differences associated with gestational age, we found no relationship between NO and CO (p = 0.18, *r* = 0.18); however, we observe a positive relationship between NO and H_2_S (p = 0.008, *r* = 0.28) and an inverse correlation between CO and H_2_S (p = 0.01, *r* = -0.33).

### Structural equation modelling

**Fig 1:**
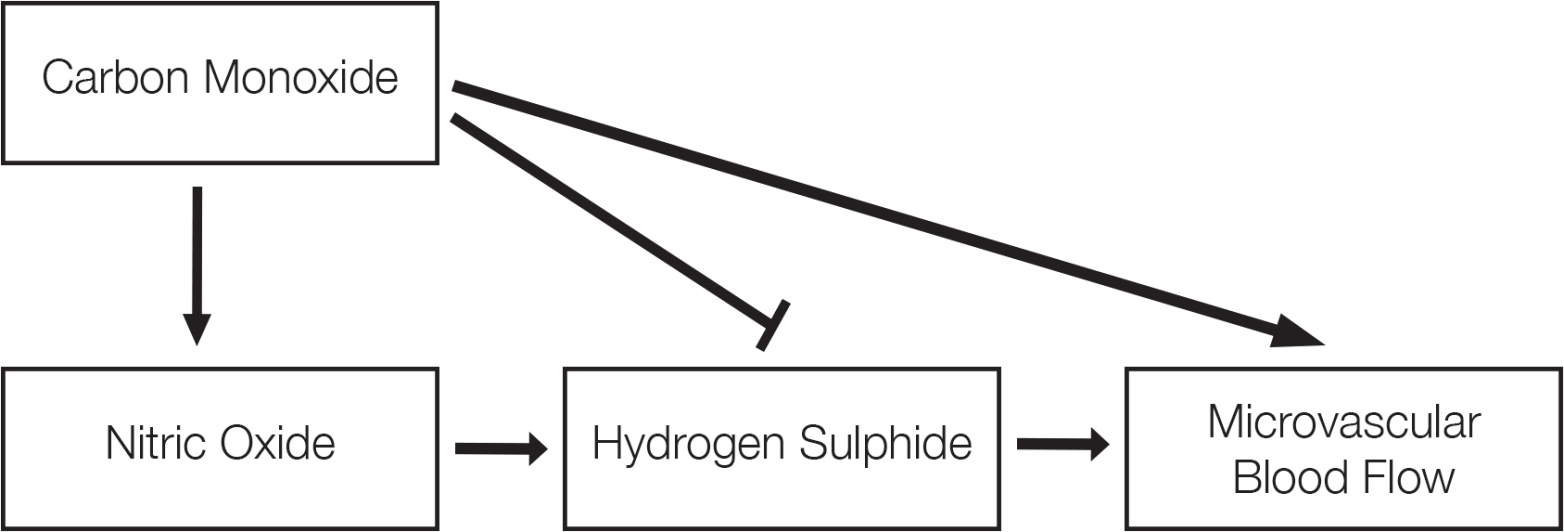
Structural equation model of predicted interactions of the gasotransmitters and their contribution to the regulation of microvascular blood flow at 24h postnatal age in the preterm human. The overall model (males and females combined) is presented and has a Goodness of Fit of χ^2^ = 1.02 and RMSEA value of 0.017 (CI 0.00–0.28). Structural equation modelling examines linear causal relationships among variables, while simultaneously accounting for measurement error. The measurement error, or variance, determined in the model is 0.66 for microvascular blood flow, 0.77 for hydrogen sulphide, 0.24 for nitric oxide and 0.07 for carbon monoxide. NO was positively correlated with H_2_S (p = 0.002, z = 3.05). There was an inverse correlation between CO and H_2_S (p = 0.18, z = -1.34). There was a significant relationship between H_2_S and microvascular blood flow (p = 0.012, z = 2.52) when the input of NO and CO to H_2_S was included in the model.

Based on the results of the present study, several theoretical models were computed (S1 Data). The overall model with the best fit (χ^2^ = 1.02; RMSEA = 0.017(CI 0.00–0.28)) is presented in Fig. 1. This model had a better Goodness of Fit in females (χ^2^ = 0.03; RMSEA<0.0001(CI 0.00–0.21)) than males (χ^2^ = 1.88; RMSEA = 0.137(CI 0.00–0.44)). In this model, NO promotes H_2_S production (overall p = 0.002, z = 3.05; males p = 0.06, z = 1.88; females p<0.0001, z = 4.53), whilst CO inhibits H_2_S (overall p = 0.18, z = -1.34; males p = 0.84, z = -0.20; females p<0.0001, z = -5.39). As described above, NO levels were higher in females than males and no differences in CO levels between sexes were observed. The net result was a slightly enhanced positive relationship of all vasodilators acting on the microvasculature in males (p = 0.006, z = 2.74) compared to the effect of H_2_S on microvascular blood flow in isolation (model constructed without inclusion of other gasotransmitters; p = 0.008, z = 2.67). In females, the model predicted a lower contribution of H_2_S on microvascular blood flow (p = 0.905, z = -0.12) compared to the effect of H_2_S in isolation (p = 0.753, z = 0.31). The model predicted covariance in the levels of NO and CO despite a lack of any direct effect of one on the other (p = 0.362, z = 0.91), this may reflect an effect of gestational age. CO had no direct effect on microvascular blood flow in the model presented, however inclusion of this pathway improved goodness of fit compared to the same model minus this interaction (overall with CO effect χ^2^ = 1.02 vs without χ^2^ = 2.34, females with CO effect χ^2^ = 0.03 vs without χ^2^ = 0.29, males with CO effect χ^2^ = 1.88 vs without χ^2^ = 2.67). Additionally, the inclusion of a direct effect of CO on microvascular blood flow increased the effect of H_2_S on blood flow in the overall model (with CO effect p = 0.012, z = 2.52 vs. without p = 0.019, z = 2.34) and in males (with CO effect p = 0.006, z = 2.74 vs. without p = 0.008, z = 2.67).

Alternate models were tested and are presented in S1 Data. None of these models had a more acceptable χ^2^ or RMSEA value and CI, thus the selection of the model presented in Fig. 1.

## Discussion

Structural equation modelling is sometimes referred to as “causal modelling”. However, a number of recent publications highlight that caution must be taken when interpreting the results as causation rather than association. Beran and Vialato [52] proposed that for causation to be determined via structural equation modelling the following criteria must be met: 1) there must be an empirical association between the variables, i.e. they are significantly correlated; 2) a common cause of the two variables must have been ruled out; 3) the two variables have a theoretical connection; and 4) that one variable precedes the other, and if the preceding variable changes, the outcome variable also changes (and not vice versa). These requirements are unlikely to be satisfied using non-experimental data, thus, causation cannot be definitively demonstrated. Rather, causal inferences that inform future experimental work may be drawn. The work presented here, and the final model proposed, in fact satisfies the majority of the criteria for causation as set out by Beran and Vialato.

Firstly, the gasotransmitters and microvascular blood flow are inter-correlated: as we have shown previously, CO [4] and H_2_S [1] were associated with higher microvascular blood flow in male preterm neonates. Contrary to our previous findings, we observed a significant, positive relationship between NO and microvascular blood flow in male preterm neonates. Furthermore, NO was positively correlated with H_2_S, whilst CO was inversely correlated with H_2_S. Thus, criteria 1 is met. Secondly, the variables have a theoretical connection (criteria 3): the gasotransmitters have known vasodilatory actions, and high microvascular blood flow in the neonate is assumed to relate to a loss of peripheral vascular tone. More specifically, a number of studies have now shown interactions between the three gasotransmitters (Table 1). Finally, criteria 4 specifies that one variable precedes the other; in the studies presented here, the testing of alternate models (see S1 Data) suggests that changes in CO and NO precede changes in H_2_S, and not vice versa; however, experimental studies need to be performed in order to confirm this directionality, especially considering the volume of experimental data that supports an effect of H_2_S on NO (Table 1 and [55]), as well as an effect of NO on H_2_S.

Thus, we present here a theoretical model, supported by our human observational studies, for the regulation of microvascular tone in the preterm newborn by the action and interaction of the gasotransmitters, which provides a construct from which future experimental studies may work in order to understand the development of circulatory compromise in this vulnerable population.

### Interactions of the Gasotransmitters and their relationship with microvascular blood flow

We observed a significant positive relationship between NO and H_2_S. Previous studies have reported that NO inhibits H_2_S production via CBS [26,27] but induces CSE expression, and consequently, H_2_S production via that pathway [25]. This may suggest that in the human preterm newborn, CSE expression is significantly modulated by NO. We have evidence from our animal model that increases in H_2_S associated with microvascular dysregulation are driven by CSE-dependent mechanisms [56]. The inhibition of CSE prevents the increased H_2_S production observed at 24h postnatal age in the preterm guinea pig pup, and CSE-dependent, but not CSE-independent H_2_S production is associated with increased microvascular blood flow. The relationship between NO and CSE/H_2_S needs to be investigated further, particularly as this appears to be associated with higher microvascular blood flow as measured by laser Doppler. Contrary to our previous findings [4], we observed a significant, positive relationship between NO and microvascular blood flow at 24h postnatal age in male neonates. One source of these differing results may be the use of different methodology—in our previous papers NO metabolites were standardised to creatinine to allow for comparisons between time points and subjects. It has been shown, however, that creatinine may not be the best molecule for this purpose in the neonate as levels change significantly in the transitional period [57,58].

In females, a lower contribution of H_2_S to microvascular tone regulation was predicted when the other gasotransmitters were added into the model. This suggests that the effect of either NO, CO, or both, negates the effect of H_2_S to such a degree that there is no net effect on vascular tone. This may be primarily due to CO, which is inversely correlated with H_2_S and may reflect an inhibitory action of CO on H_2_S, in line with published reports that have demonstrated that CO decreases the production and action of H_2_S [34,35,36]. This is of particular interest in this cohort, as females and males had comparable levels of CO, suggesting some protective role of this molecule against inappropriate vasodilation in the female. The findings of our current study are discrepant with our previous studies, which showed that males had higher levels of CO and that this was associated with inappropriate peripheral microvascular dilatation and physiological instability in the first few days of life [4]. There are a number of possible explanations for these differences. Firstly, the infants in our original studies were younger (median age 1 week older in the present study, with neonates up to 35 weeks included compared to an upper age of 32 weeks in the previous study) and therefore, sicker, than the neonates in the present study. Secondly, there was a much higher rate of antenatal glucocorticoid exposure in the present study (74% in the current study compared to 59% in the previous study).

### Limitations and future research

The present study does not provide direct confirmation of the mechanisms of action, the expression of gasotransmitter-producing enzyme/s or feedback of the gaseous molecule on the producing and/or releasing pathways. Rather, the aim of this study was to establish a theoretical model of gasotransmitter interactions in the preterm newborn, and the potential effect of these interactions on microvascular blood flow. Given the evidence of interactions between the three gasotransmitters in the preterm newborn population presented here, we propose future mechanistic studies should not focus solely on one of these gasotransmitters as driving dysfunction, but rather investigate the interactions among CO, NO and H_2_S in this context.

It is not possible to experimentally test these interactions within the sick human preterm infant population studied here, however the results of the present study can be used to inform future studies in relevant animal models [59,60,61] in order to elucidate the mechanisms underlying these correlations.

Future research should also investigate the mechanisms that give rise to the different interactions and effects of the gasotransmitters in male versus female preterm neonates. As many of the steroid hormone receptors (such as those for progesterone, estrogen and testosterone) are located within the endothelium and smooth muscle layers of blood vessels, the sex hormones may have influence over these vasoactive substances and the downstream signalling mechanisms involved in microvascular dilatation in a sex-specific manner.

We hypothesised that rates of antenatal glucocorticoids may contribute to the differences in CO levels and effect on blood flow observed in the present study compared to our previous studies in a similar cohort [4]. Glucocorticoids, such as antenatally administered betamethasone can modulate blood pressure, vascular reactivity and the production and action of vasoconstrictors and vasodilators, such as the gasotransmitters [62,63]. In mice, administration of glucocorticoids reduces eNOS levels (through decreased transcription and increased degradation) in aorta, liver and kidney [64,65,66,67]. Dexamethasone is known to reduce the release of NO from the endothelium and completely suppresses the inducible form of NOS [68,69,70,71]. Dexamethasone also downregulates HO-1 expression in models of systemic inflammation [72] and suppresses CSE expression, reducing H_2_S production, both directly through regulation of transcription and through inhibition of NO production, which is known to drive CSE expression [24]. Glucocorticoids are also known to decrease other vasodilators, including prostaglandins and enhance the effects of vasoconstrictors such as Angiotensin II [73,74] and norepinephrine [63,75,76]. The effect of glucocorticoids on the levels of individual vasoactive molecules, and overall vascular tone regulation, needs to be studied further in order to determine if antenatal glucocorticoid exposure effects gasotransmitters production and/or action in the preterm newborn. This is particularly relevant in the context of sex differences in gasotransmitters-related regulation of vascular tone, as it is well characterised that males and females metabolise and respond to glucocorticoid exposure differently [48,77].

Future studies should also consider the effect of a range of other vasoactive inputs, such as the sympathetic nervous system and the renin-angiotensin system and the newly identified fourth gasotransmitter, ammonium [78]. The action of these pathways and their interaction with the gasotransmitter system presented here may contribute to overall vascular tone regulation in this vulnerable population.

## Conclusions

We identified significant correlations between the gasotransmitters NO, CO and H_2_S and microvascular blood flow in preterm neonates. This allowed us to produce a theoretical model for the regulation of microvascular tone in the preterm newborn by the action and interaction of the gasotransmitters. The results of the present study suggest that CO may confer some protective advantage in the female preterm neonate whilst in the male neonate, H_2_S production may be aberrantly modulated by NO, likely through changes in CSE expression. This hypothesis is supported by the results of the present study, previous studies by others (see Table 1) and those of ourselves—we have shown that CSE production is upregulated in the preterm newborn male and that H_2_S produced via CSE (but not CSE-independent pathways) correlates with microvascular tone dysregulation [56]. The relationship between NO and CSE/H_2_S is associated with higher microvascular blood flow and may be of particular interest given the wealth of literature surrounding an interaction between these two molecules (and their production pathways); further work is required in order to confirm this. We present a theoretical model built on observations within a human population which provides evidence of gasotransmitters interactions in the preterm newborn. This model provides a framework for establishing and testing current and future mechanistic hypotheses within this population.

## Supporting Information

S1 Data Alternate Structural Equation Models. Alternate models were tested and are presented here. As in the manuscript, the interaction between the three gasotransmitters and their individual and combined effects on microvascular blood flow were assessed. All models were manually constructed and presented here for our three input (NO, CO and H_2_S) and one output (microvascular blood flow) variables. These models were then tested and assessed for suitability by χ^2^ Goodness of Fit and root mean square error of approximation (RMSEA). Lower χ^2^ values represent a better predicted model, whilst an RMSEA of below 0.06 shows a good fit. RMSEA also allows for calculation of a confidence interval (CI) around the predictive power of the model. None of these models had a more acceptable χ^2^ or RMSEA value and CI, thus the selection of the model presented within the manuscript.
(PDF)
