# Supplementary material for: Interactions of the Gasotransmitters Contribute to Microvascular Tone (Dys)regulation in the Preterm Neonate
Source: PLoS One. 2015 Mar 25;10(3):e0121621. doi: 10.1371/journal.pone.0121621 (PMC4373676; doi:10.1371/journal.pone.0121621)
Supplement: S1 Data — Alternate models were tested and are presented here. As in the manuscript, the interaction between the three gasotransmitters and their individual and combined effects on microvascular blood flow were assessed. All models were manually constructed and presented here for our three input (NO, CO and H2S) and one output (microvascular blood flow) variables. These models were then tested and assessed for suitability by χ2 Goodness of Fit and root mean square error of approximation (RMSEA). Lower χ2 values represent a better predicted model, whilst an RMSEA of below 0.06 shows a good fit. RMSEA also allows for calculation of a confidence interval (CI) around the predictive power of the model. None of these models had a more acceptable χ2 or RMSEA value and CI, thus the selection of the model presented within the manuscript. (PDF) [file pone.0121621.s001.pdf]

Supplementary Data

Alternate structural equation models are presented here.

As in the manuscript, the interaction between the three gasotransmitters and their individual and combined effects on microvascular blood flow were assessed. All models were manually constructed and presented here for our three input (NO, CO and H_2S) and one output (microvascular blood flow) variables. These models were then tested and assessed for suitability by χ^2 Goodness of Fit and root mean square error of approximation (RMSEA). Lower χ^2 values represent a better predicted model, whilst an RMSEA of below 0.06 shows a good fit. RMSEA also allows for calculation of a confidence interval (CI) around the predictive model of the model.

For each model presented, the figure represents the overall interaction (males and females combined). Information regarding sex differences can be found in the tables below each figure. Results for χ^2 and RMSEA(CI) are presented in each table. Negative z values indicate an inverse correlation (or inhibitory effect) whilst positive z values indicate a positive correlation (stimulatory or permissive effect).

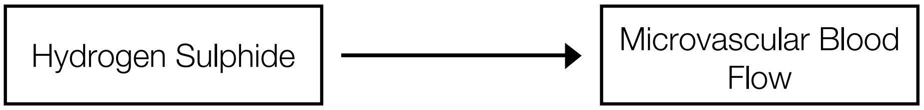

	Female	Male	Overall
H_2S → Flow	p=0.75 (z=0.31)	p=0.008 (z=2.67)	p=0.019 (z=2.34)
χ^2	<0.0001	<0.0001	<0.0001
RMSEA	0.00	0.00	0.00

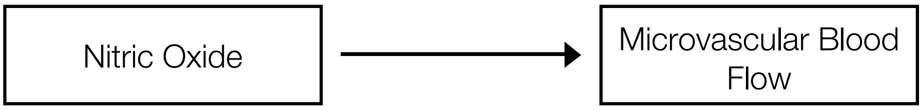

	Female	Male	Overall
NO → Flow	p=0.99 (z=-0.01)	p=0.05 (z=1.99)	p=0.08 (z=1.78)
χ^2	<0.0001	<0.0001	<0.0001
RMSEA	0.00	0.00	0.00

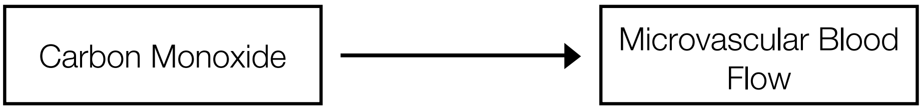

	Female	Male	Overall
CO $\rightarrow$ Flow	p=0.95 (z=0.06)	p=0.20 (z=1.28)	p=0.38 (z=0.89)
χ^2	<0.0001	<0.0001	<0.0001
RMSEA	0.00	0.00	0.00

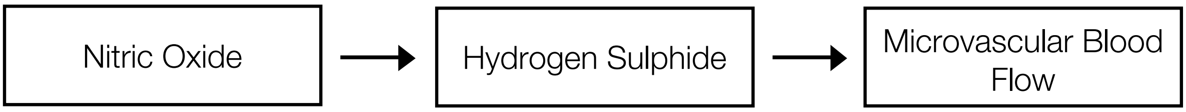

	Female	Male	Overall
NO → H_2S	p=0.004 (z=2.91)	p=0.06 (z=1.87)	p=0.005 (z=2.82)
H_2S → Flow	p=0.75 (z=0.31)	p=0.008 (z=2.67)	p=0.02 (z=2.34)
χ^2	0.03	2.0	1.41
RMSEA	0.0 (0.0-0.21)	0.15 (0.0-0.45)	0.07 (0.0-0.3)

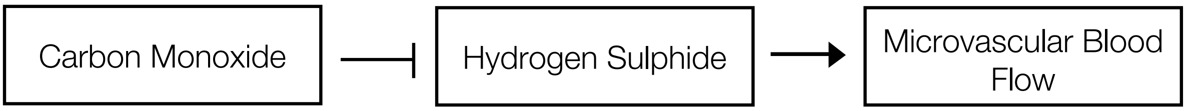

	Female	Male	Overall
CO → H_2S	p=0.13 (z=-1.51)	p=0.81 (z=-0.24)	p=0.39 (z=-0.86)
H_2S → Flow	p=0.75 (z=0.31)	p=0.008 (z=2.67)	p=0.02 (z=2.34)
χ^2	0.004	1.81	1.11
RMSEA	0.00	0.13 (0.00-0.44)	0.04 (0.00-0.28)

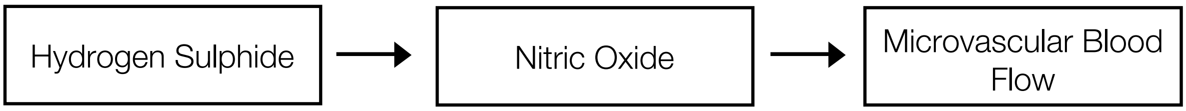

	Female	Male	Overall
H_2S → NO	p=0.004 (z=2.91)	p=0.06 (z=1.87)	p=0.005 (z=2.82)
NO → Flow	p=0.98 (z=-0.01)	p=0.05 (z=1.99)	p=0.08 (z=1.99)
χ^2	0.12	4.83	3.61
RMSEA	0.00 (0.00-0.29)	0.29 (0.08-0.56)	0.17 (0.00-0.37)

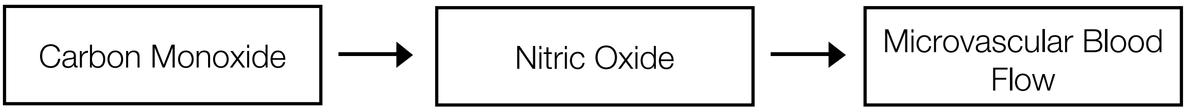

	Female	Male	Overall
CO → NO	p=0.22 (z=1.23)	p=0.89 (z=0.14)	p=0.34 (z=0.95)
NO → Flow	p=0.99 (z=-0.01)	p=0.05 (z=1.99)	p=0.08 (z=1.78)
χ^2	<0.0001	1.56	0.56
RMSEA	0.00	0.11 (0.00-0.42)	0.00 (0.00-0.25)

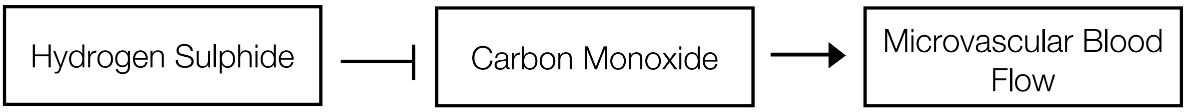

	Female	Male	Overall
H_2S → CO	p=0.19 (z=-1.32)	p=0.87 (z=0.17)	p=0.54 (z=-0.61)
CO → Flow	p=0.83 (z=-0.21)	p=0.20 (z=1.29)	p=0.51 (z=0.66)
χ^2	0.06	6.94	5.99
RMSEA	0.00 (0.00-0.25)	0.36 (0.14-0.62)	0.24 (0.08-0.43)

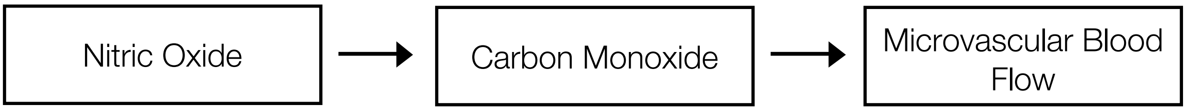

	Female	Male	Overall
NO → CO	p=0.22 (z=1.22)	p=0.79 (z=0.26)	p=0.32 (z=1.00)
CO → Flow	p=0.98 (z=-0.02)	p=0.19 (z=1.30)	p=0.35 (z=0.94)
χ^2	<0.0001	3.77	2.82
RMSEA	0.00	0.24 (0.00-0.52)	0.14 (0.00-0.35)

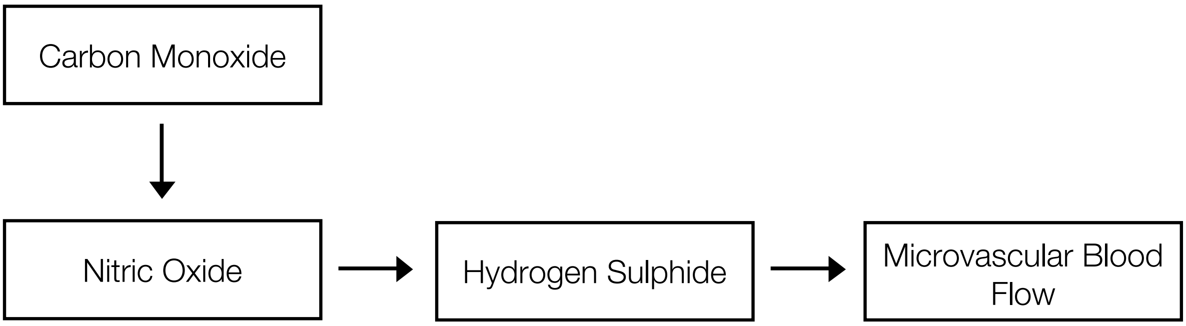

	Female	Male	Overall
CO → NO	p=0.22 (z=1.23)	p=0.89 (z=0.14)	p=0.34 (z=0.95)
NO → H_2S	p=0.004 (z=2.91)	p=0.06 (z=1.87)	p=0.005 (z=2.82)
H_2S → Flow	p=0.75 (z=0.31)	p=0.008 (z=2.67)	p=0.02 (z=2.34)
χ^2	7.39	3.84	4.03
RMSEA	0.18 (0.00-0.36)	0.08 (0.00-0.27)	0.06 (0.00-0.2)

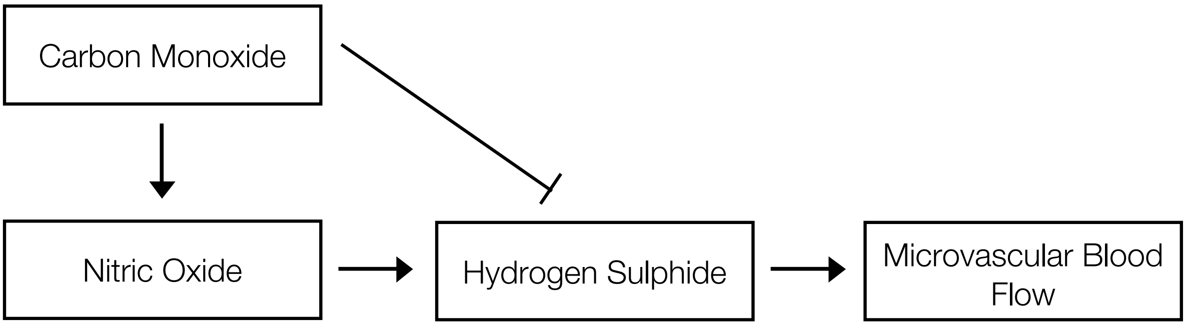

	Female	Male	Overall
CO → NO	p=0.38 (z=0.89)	p=0.87 (z=0.16)	p=0.31 (z=1.02)
CO → H_2S	p<0.0001 (z=-5.21)	p=0.75 (z=-0.31)	p=0.15 (z=-1.43)
NO → H_2S	p<0.0001 (z=4.46)	p=0.06 (z=1.88)	p=0.002 (z=3.06)
H_2S → Flow	p=0.75 (z=0.31)	p=0.008 (z=2.67)	p=0.02 (z=2.34)
χ^2	0.29	3.74	2.16
RMSEA	0.00 (0.00-0.16)	0.14 (0.00-0.35)	0.03 (0.00-0.21)

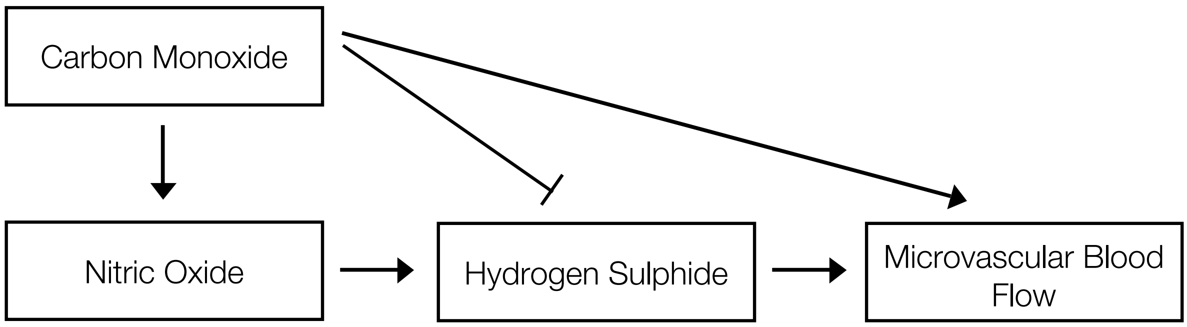

	Female	Male	Overall
CO → NO	p=0.37 (z=0.89)	p=0.77 (z=0.30)	p=0.29 (z=1.06)
CO → H_2S	p<0.0001 (z=-5.39)	p=0.84 (z=-0.20)	p=0.18 (z=-1.34)
CO → Flow	p=0.60 (z=-0.52)	p=0.16 (z=1.40)	p=0.28 (z=1.08)
NO → H_2S	p<0.0001 (z=4.53)	p=0.06 (z=1.88)	p=0.002 (z=3.05)
H_2S → Flow	p=0.91 (z=-0.12)	p=0.006 (z=2.74)	p=0.01 (z=2.52)
χ^2	0.03	1.88	1.02
RMSEA	0.00 (0.00-0.21)	0.14 (0.00-0.44)	0.02 (0.00-0.28)

This is the model presented in the manuscript and is included here for ease of comparison

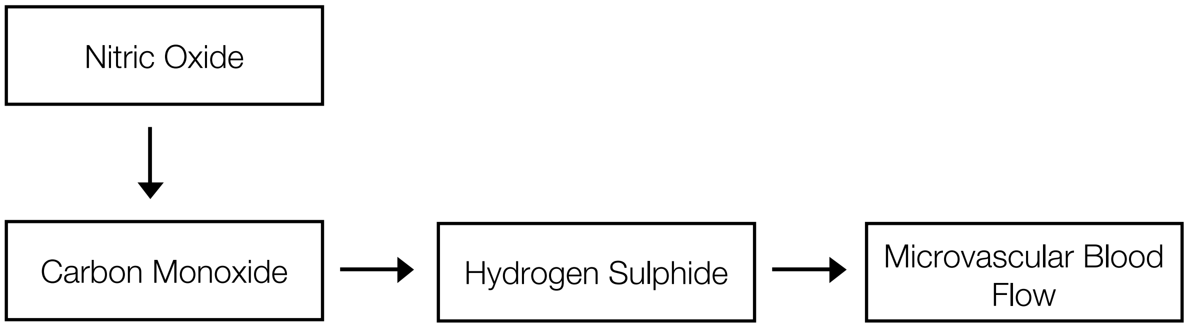

	Female	Male	Overall
NO → CO	p=0.38 (z=0.89)	p=0.89 (z=0.14)	p=0.35 (z=0.93)
CO → H_2S	p=0.30 (z=1.05)	p=0.81 (z=-0.24)	p=0.41 (z=-0.82)
H_2S → Flow	p=0.75 (z=0.31)	p=0.008 (z=2.67)	p=0.02 (z=2.34)
χ^2	14.19	7.16	10.99
RMSEA	0.30 (0.15-0.46)	0.17 (0.00-0.34)	0.17 (0.07-0.29)

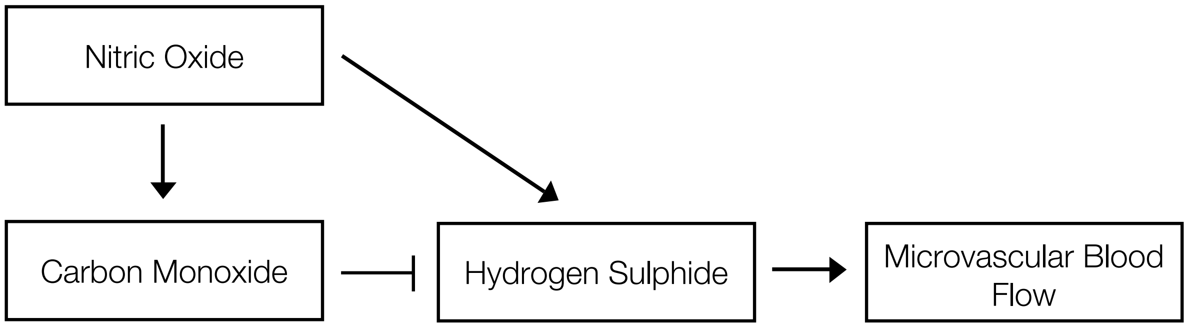

	Female	Male	Overall
NO → CO	p=0.36 (z=0.92)	p=0.87 (z=0.16)	p=0.31 (z=1.02)
NO → H_2S	p<0.0001 (z=4.46)	p=0.06 (z=1.88)	p=0.002 (z=3.06)
CO → H_2S	p<0.0001 (z=-5.21)	p=0.75 (z=-0.31)	p=0.15 (z=-1.43)
H_2S → Flow	p=0.75 (z=0.31)	p=0.008 (z=2.67)	p=0.02 (z=2.34)
χ^2	0.29	3.74	2.16
RMSEA	0.00 (0.00-0.16)	0.14 (0.00-0.35)	0.03 (0.00-0.21)

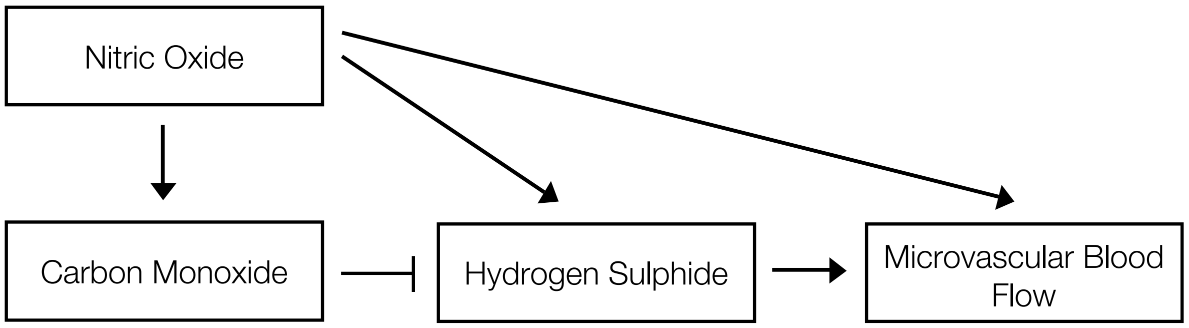

	Female	Male	Overall
NO → CO	p=0.36 (z=0.92)	p=0.87 (z=0.16)	p=0.31 (z=1.02)
NO → H_2S	p<0.0001 (z=4.46)	p=0.06 (z=1.88)	p=0.002 (z=3.06)
NO → Flow	p=0.87 (z=-0.16)	p=0.15 (z=1.43)	p=0.23 (z=1.19)
CO → H_2S	p<0.0001 (z=-5.21)	p=0.75 (z=-0.31)	p=0.15 (z=-1.43)
H_2S → Flow	p=0.73 (z=0.35)	p=0.02 (z=2.26)	p=0.06 (z=1.92)
χ^2	0.27	1.75	1.75
RMSEA	0.27 (0.00-0.32)	0.13 (0.00-0.43)	0.00 (0.00-0.26)

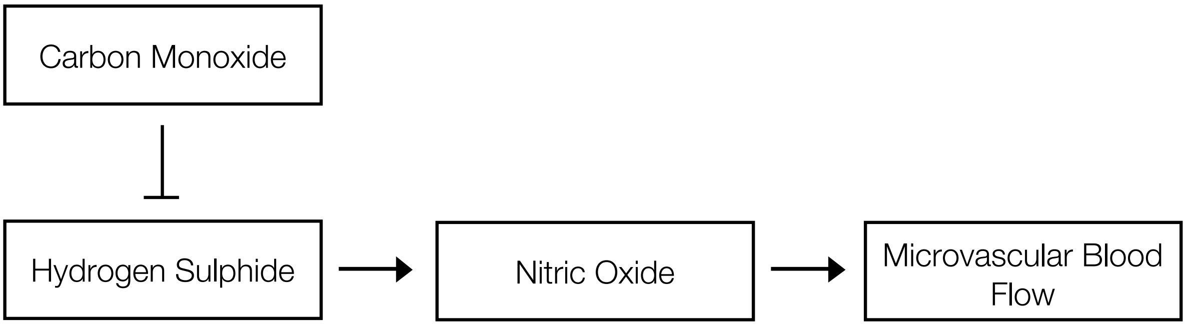

	Female	Male	Overall
CO → H_2S	p=0.31 (z=-1.51)	p=0.81 (z=-0.24)	p=0.39 (z=-0.86)
H_2S → NO	p=0.004 (z=2.91)	p=0.06 (z=1.87)	p=0.005 (z=2.82)
NO → Flow	p=0.99 (z=-0.01)	p=0.05 (z=1.99)	p=0.08 (z=1.78)
χ^2	7.30	6.64	6.42
RMSEA	0.18 (0.00-0.36)	0.16 (0.00-0.34)	0.11 (0.00-0.24)

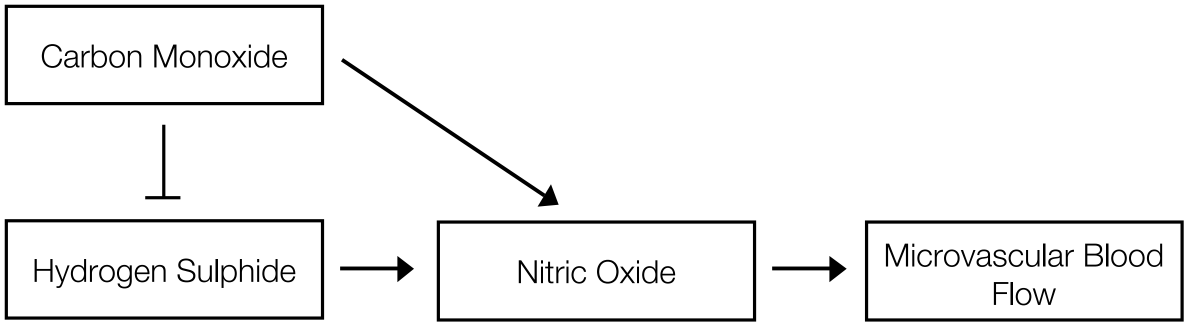

	Female	Male	Overall
CO → H_2S	p<0.0001 (z=-3.69)	p=0.78 (z=-0.27)	p=0.25 (z=-1.16)
CO → NO	p=0.002 (z=3.10)	p=0.81 (0.25)	p=0.15 (z=1.45)
H_2S → NO	p<0.0001 (z=3.52)	p=0.06 (z=1.88)	p=0.003 (z=3.02)
NO → Flow	p=0.99 (z=-0.01)	p=0.05 (z=1.99)	p=0.08 (z=1.78)
χ^2	0.39	6.58	4.37
RMSEA	0.00 (0.00-0.18)	0.22 (0.05-0.42)	0.12 (0.00-0.26)

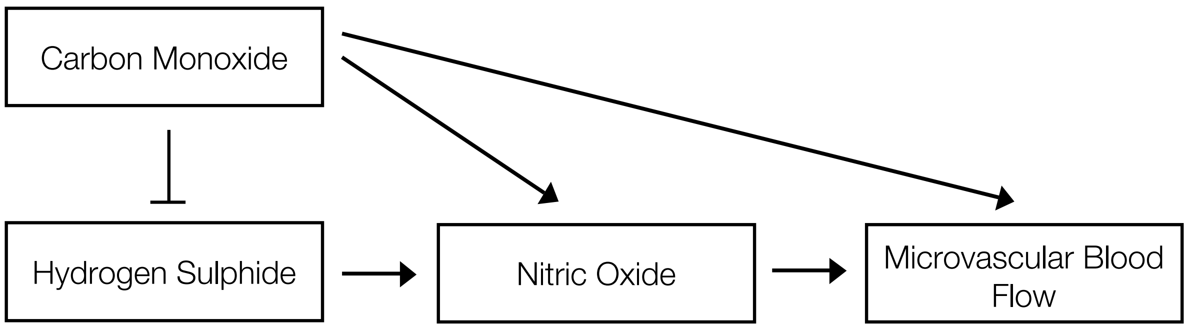

	Female	Male	Overall
CO → H_2S	$p<0.0001$ (z-3.78)	p=0.87 (z=0.16)	p=0.32 (z=-0.99)
CO → NO	p=0.001 (z=3.18)	p=0.88 (z=0.15)	p=0.16 (z=1.39)
CO → Flow	p=0.55 (z=-0.60)	p=0.20 (z=1.28)	p=0.74 (z=0.33)
H_2S → NO	$p<0.0001$ (z=3.58)	p=0.06 (z=1.86)	p=0.003 (z=2.98)
NO → Flow	p=0.97 (z=0.04)	p=0.05 (z=1.98)	p=0.09 (z=1.72)
χ^2	0.04	5.10	4.26
RMSEA	0.00 (0.00-0.23)	0.30 (0.09-0.57)	0.19 (0.04-0.39)

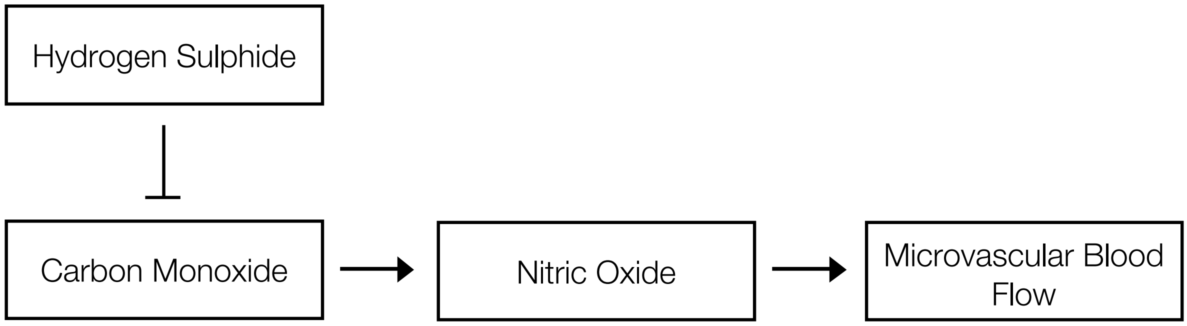

	Female	Male	Overall
H_2S → CO	p=0.34 (z=-0.96)	p=0.81 (z=-0.24)	p=0.41 (z=-0.82)
CO → NO	p=0.39 (z=0.86)	p=0.89 (z=0.14)	p=0.36 (z=0.92)
NO → Flow	p=0.99 (z=-0.01)	p=0.05 (z=1.99)	p=0.08 (z=1.78)
χ^2	14.29	10.00	13.20
RMSEA	0.30 (0.15-0.46)	0.22 (0.08-0.38)	0.19 (0.10-0.31)

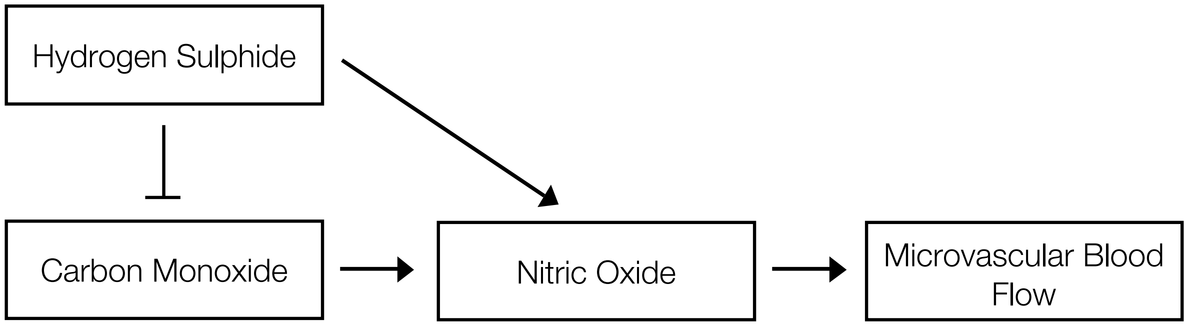

	Female	Male	Overall
H_2S → CO	p=0.02 (z=-2.35)	p=0.78 (z=-0.27)	p=0.26 (z=-1.12)
H_2S → NO	p<0.0001 (z=3.52)	p=0.06 (z=1.88)	p=0.003 (z=3.02)
CO → NO	p=0.002 (z=3.10)	p=0.81 (z=0.25)	p=0.15 (z=1.45)
NO → Flow	p=0.99 (z=-0.01)	p=0.05 (z=1.99)	p=0.08 (z=1.78)
χ^2	0.39	6.58	4.37
RMSEA	0.00 (0.00-0.18)	0.22 (0.05-0.42)	0.12 (0.00-0.26)

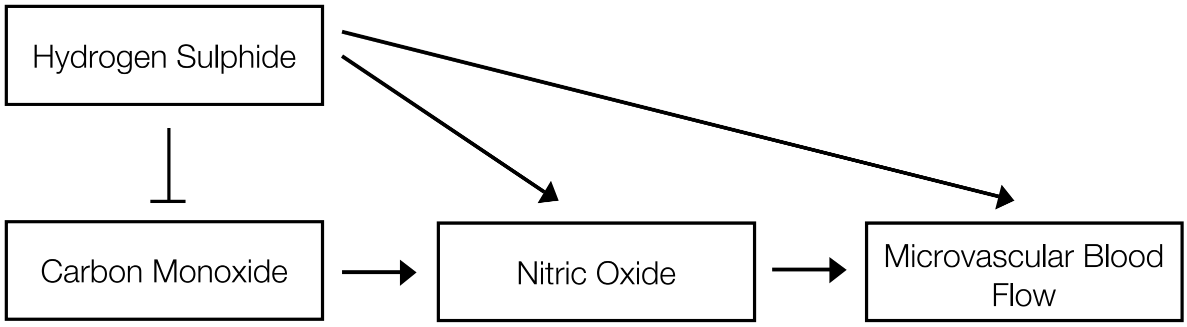

	Female	Male	Overall
H_2S → CO	p=0.02 (z=-2.35)	p=0.78 (z=-0.27)	p=0.26 (z=-1.12)
H_2S → NO	p<0.0001 (z=3.52)	p=0.06 (z=1.88)	p=0.003 (z=3.02)
H_2S → Flow	p=0.73 (z=0.35)	p=0.02 (z=2.26)	p=0.06 (z=1.92)
CO → NO	p=0.002 (z=3.10)	p=0.81 (z=0.25)	p=0.15 (z=1.43)
NO → Flow	p=0.87 (z=-0.16)	p=0.15 (z=1.43)	p=0.23 (z=1.19)
χ^2	0.27	1.75	1.75
RMSEA	0.00 (0.00-0.32)	0.13 (0.00-0.43)	0.00 (0.00-0.26)

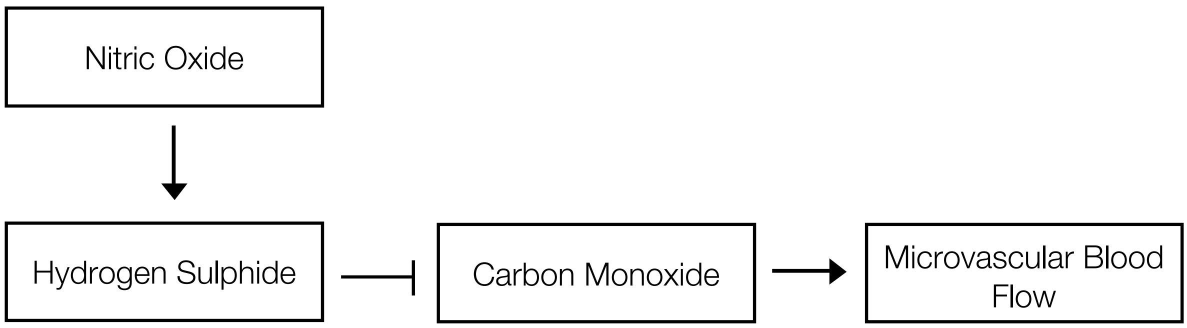

	Female	Male	Overall
NO → H_2S	p=0.004 (z=2.91)	p=0.06 (z=1.87)	p=0.005 (z=2.82)
H_2S → CO	p=0.19 (z=-1.32)	p=0.87 (z=0.17)	p=0.54 (z=-0.61)
CO → Flow	p=0.83 (z=-0.21)	p=0.20 (z=1.29)	p=0.51 (z=0.66)
χ^2	7.26	8.93	9.10
RMSEA	0.18 (0.00-0.36)	0.21 (0.06-0.37)	0.15 (0.04-0.27)

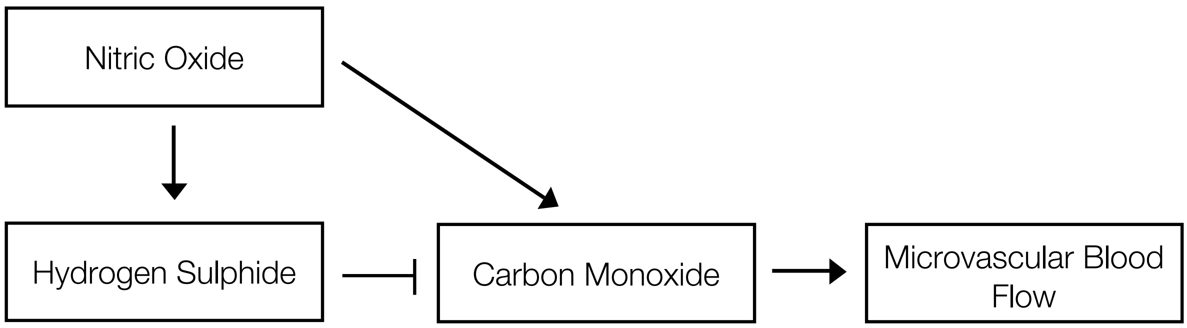

	Female	Male	Overall
NO → H_2S	p=0.004 (z=2.91)	p=0.06 (z=1.87)	p=0.004 (z=2.82)
NO → CO	p=0.004 (z=2.86)	p=0.83 (z=0.22)	p=0.16 (z=1.40)
H_2S → CO	p=0.003 (z=-2.93)	p=0.94 (z=0.08)	p=0.25 (z=-1.15)
CO → Flow	p=0.55 (z=-0.60)	p=0.02 (z=1.28)	p=0.60 (z=0.53)
χ^2	0.04	8.08	7.19
RMSEA	0.00	0.27 (0.11-0.46)	0.17 (0.05-0.31)

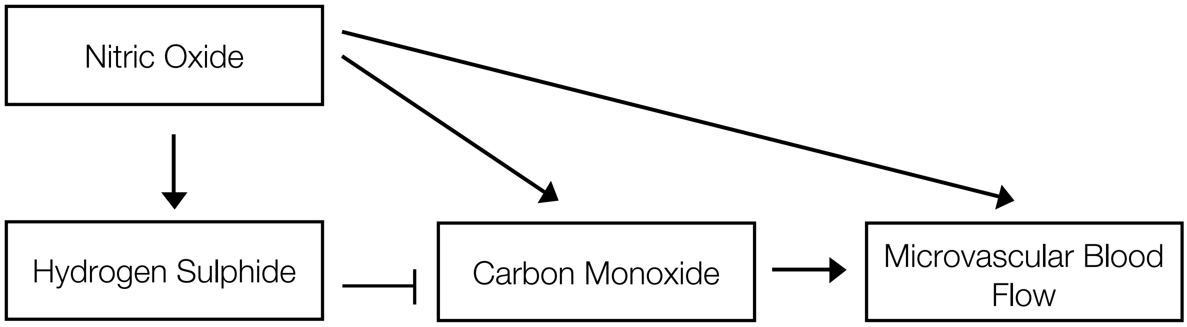

	Female	Male	Overall
NO → H_2S	p=0.004 (z=2.82)	p=0.06 (z=1.87)	p=0.005 (z=2.82)
NO → CO	p=0.004 (z=2.86)	p=0.88 (z=0.15)	p=0.16 (z=1.39)
NO → Flow	p=0.97 (z=0.04)	p=0.05 (z=1.98)	p=0.09 (z=1.72)
H_2S → CO	p=0.003 (z=-2.93)	p=0.90 (z=0.12)	p=0.23 (z=-1.20)
CO → Flow	p=0.55 (z=-0.60)	p=0.20 (z=1.28)	p=0.74 (z=0.33)
χ^2	0.04	5.10	4.26
RMSEA	0.00 (0.00-0.23)	0.30 (0.09-0.57)	0.19 (0.04-0.39)

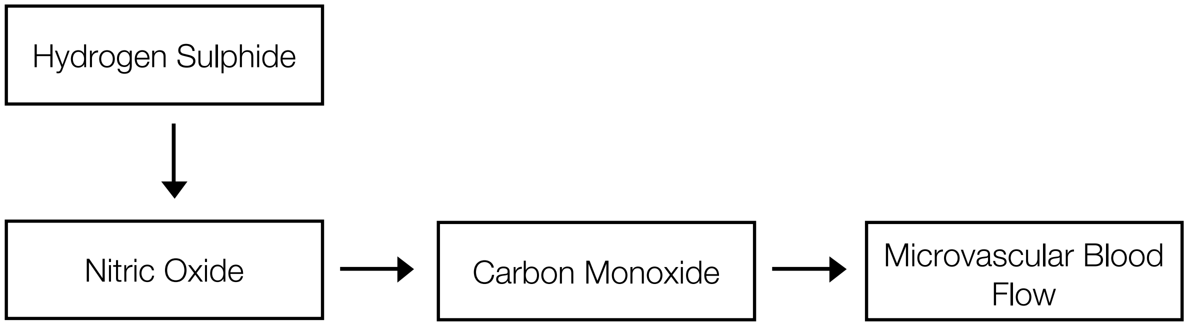

	Female	Male	Overall
H_2S → NO	p=0.004 (z=2.91)	p=0.06 (z=1.87)	p=0.005 (z=2.82)
NO → CO	p=0.22 (z=1.22)	p=0.79 (z=0.26)	p=0.32 (z=1.00)
CO → Flow	p=0.99 (z=-0.02)	p=0.19 (z=1.30)	p=0.35 (z=0.94)
χ^2	7.48	8.89	8.49
RMSEA	0.19 (0.00-0.36)	0.20 (0.06-0.37)	0.14 (0.03-0.26)

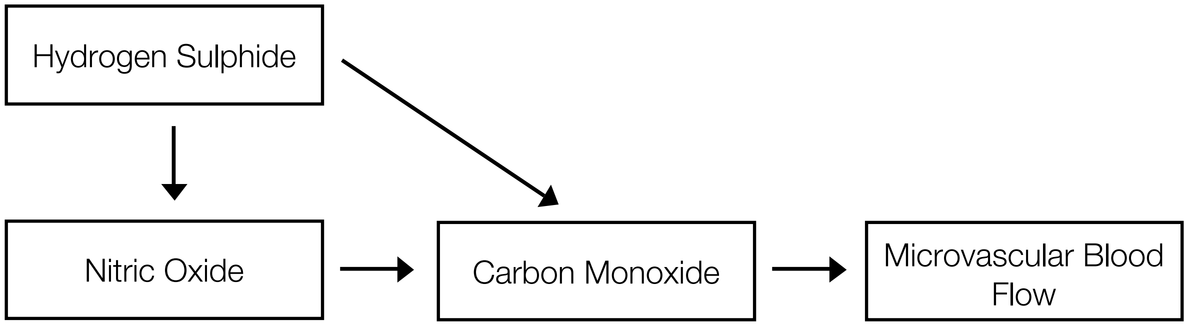

	Female	Male	Overall
H_2S → NO	p=0.004 (z=2.91)	p=0.06 (z=1.87)	p=0.005 (z=2.82)
H_2S → CO	p=0.003 (z=-2.93)	p=0.94 (z=0.08)	p=0.25 (z=-1.15)
NO → CO	p=0.004 (z=2.86)	p=0.83 (z=0.22)	p=0.16 (z=1.40)
CO → Flow	p=0.55 (z=-0.60)	p=0.20 (z=1.28)	p=0.60 (z=0.53)
χ^2	0.04	8.88	7.19
RMSEA	0.00	0.27 (0.11-0.46)	0.17 (0.05-0.31)

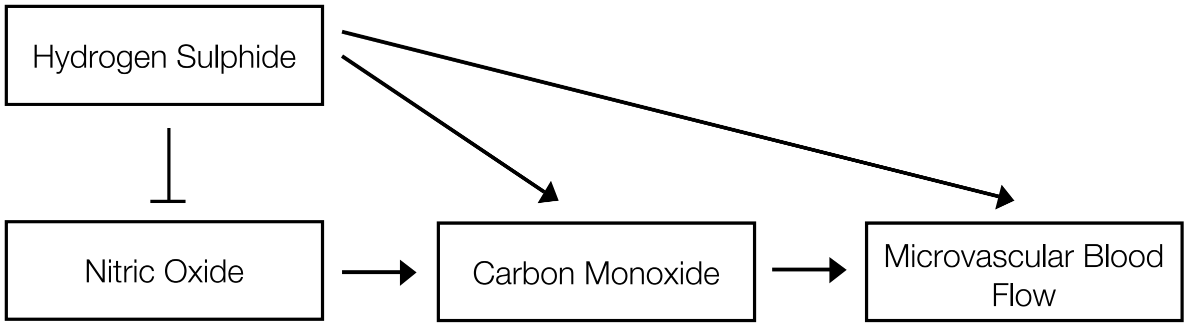

	Female	Male	Overall
H_2S → NO	p=0.005 (z=2.91)	p=0.06 (z=1.87)	p=0.005 (z=2.82)
H_2S → CO	p=0.004 (z=-2.91)	p=0.84 (z=-0.20)	p=0.19 (z=-1.31)
H_2S → Flow	p=0.91 (z=-0.12)	p=0.006 (z=2.74)	p=0.01 (z=2.52)
NO → CO	p=0.004 (z=2.86)	p=0.74 (z=0.34)	p=0.15 (z=1.46)
CO → Flow	p=0.60 (z=-0.52)	p=0.16 (z=1.40)	p=0.28 (z=1.08)
χ^2	0.03	1.88	1.02
RMSEA	0.00 (0.00-0.21)	0.14 (0.00-0.44)	0.02 (0.00-0.28)
